# Population analysis of xenobiotic metabolizing genes in South Brazilian Euro and Afro-descendants

**DOI:** 10.1590/S1415-47572009005000087

**Published:** 2009-12-01

**Authors:** Marcos Euzébio Maciel, Fausto Koga Oliveira, Gustavo Bonfim Propst, Maria da Graça Bicalho, Iglenir João Cavalli, Enilze Maria de Souza Fonseca Ribeiro

**Affiliations:** Departamento de Genética, Universidade Federal do Paraná, Curitiba, PRBrazil

**Keywords:** CYP, GST, population study

## Abstract

Individual variability in xenobiotic metabolism has been associated with susceptibility to developing complex diseases. Genes involved in xenobiotic metabolism have been evaluated in association studies; the difficulty of obtaining accurate gene frequencies in mixed populations makes interpretation of the results difficult. We sought to estimate population parameters for the cytochrome P450 and glutathione S-transferase gene families, thus contributing to studies using these genes as markers. We describe the frequencies of six genes (*CYP1A1*, *CYP2D6*, *CYP2E1*, *GSTM1*, *GSTT1*, and *GSTP1*) and estimate population parameters in 115 Euro-descendants and 196 Afro-descendants from Curitiba, South of Brazil. PCR-based methods were used for genotyping, and statistical analysis were performed by AMOVA with ARLEQUIN software. The mutant allele frequencies in the Afro-descendants and Euro-descendants, respectively, were: CYP1A1*2A = 30.1% and 15.2%; CYP2D6*4 = 14.5% and 21.5%; CYP2E1*5B = 7.9% and 5%; GSTP1*B = 37.8% and 28.3%. The null genotype frequencies were: GSTM1*0 = 36.8% and 46.1%; GSTT1*0 = 24.2% and 17.4%.

Genetic marker studies assessing individual backgrounds from specific populations can provide information on gene flow, evolutionary history, and population dispersions, and can also help in the prediction of risks for particular diseases. Based on these studies, pharmacogenetic data have shown significant inter- and intra-population differences in the metabolism, efficiencies, and toxicities of several types of drugs. These findings have important implications for the management and treatment of human diseases (Kittles and Weiss, 2003).

Many different enzyme families are involved in xenobiotic metabolism, including cytochrome P450 (CYPs) in phase I, as well as glutathione S-transferases (GSTs) and N-acetyl-transferases (NATs) in phase II (Autrup, 2000). Several genes of the CYP family have been studied in many populations (*e.g.*, Europeans, Africans, Asians, and their mixed descendants) in case-control studies of complex diseases. With regard to cancers, these studies focus primarily on lung, breast, and head and neck tumors (Olshan *et al.*, 2000; Gajecka *et al.*, 2005; Yang *et al.*, 2005; Leichsenring *et al.*, 2006; Losi-Guembarovski *et al.*, 2008; Torresan *et al.*, 2008; Varela-Lema *et al.*, 2008).

Variants of GSTs enzymes have been extensively studied and were found to be associated with several types of neoplasias in different populations, such as Europeans and Euro-descendants (Park *et al.*, 2000; Geisler and Olshan, 2001; Raimondi *et al.*, 2005; Leichsenring *et al.*, 2006; Losi-Guembarovski *et al.*, 2008; Torresan *et al.*, 2008), Africans and Afro-descendants (Dandara *et al.*, 2002; Enokida *et al.*, 2005), and Asians (Yang *et al.*, 2005). Other studies involving genes of xenobiotic metabolism have been performed in order to describe the frequency of the mutant alleles and genotypes in different healthy populations (Garte *et al.*, 2001; Gaspar *et al.*, 2002; Menoyo *et al.*, 2006). Some studies carried out in the Brazilian population described mutant allele and genotype frequencies in several regions (Arruda *et al.*, 1998; Gattás and Soares Viera, 2000; Gaspar *et al.*, 2002; Losi-Guembarovski *et al.*, 2002; Rossini *et al.*, 2002; Amorim *et al.*, 2004; Gattás *et al.*, 2004; Hatagima *et al.*, 2004; Kvitko *et al.*, 2006; Rossini *et al.*, 2006).

In the present report, two distinct groups (Euro-descendants and Afro-descendants) from Curitiba in the South of Brazil were analyzed in order to describe the frequency of six metabolic genes (*CYP1A1,CYP2E1*, *CYP2D6*, *GSTM1*, *GSTT1*, and *GSTP1*). The group of Euro-descendants was comprised of 115 healthy individuals (49 males and 66 females) with an average age of 42.6 ± 7.3 years. The group of Afro-descendants was comprised of 196 healthy individuals (123 males and 73 females) with an average age of 33.4 ± 8.6 years. The ethnic differentiation from these groups was determined through a survey with self-declared information from the individuals that was attached to the Informed Consent agreement. The blood samples were collected in the Hematology and Hemotherapy Center of Paraná State (HEMEPAR), a center for blood donation, by the staff of the Immunogenetics and Histocompatibility Laboratory (LIGH). Genomic DNA was isolated from peripheral white blood cells from all individuals and sampled by a salting out procedure (Bignon and Fernandez-Viña, 1997). Polymerase chain reaction (PCR) primers were designed according to the Genome Data Bank. The genotyping of CYP1A1*2A, CYP2D6*4, CYP2E1*5B, and GSTP1*B was performed by PCR RFLP according to the following protocols, respectively: Carstensen *et al.* (1993), Sobtia *et al.* (2005), Kato *et al.* (1992), and Harries *et al.* (1997). GSTM1*0 and GSTT1*0 genotyping was performed by PCR multiplex according to the protocol described by Abdel-Rahman *et al.* (1996).

The allele frequencies of the CYP1A1*2A, CYP2D6*4, CYP2E*5B, GSTP1*B and the null genotypes GSTM1*0 and GSTT1*0 were obtained by direct counting. The Chi-square test was used to: 1) compare the frequencies of dominant and recessive genotypes of the genes GSTM1*0 and GSTT1*0 in individuals of the Euro and Afro-descendant groups, 2) verify whether the genes CYP1A1*2A, CYP2D6*4, CYP2E*5B, and GSTP1*B were in Hardy-Weinberg equilibrium (HWE), and 3) compare the frequencies of the mutant allele of these genes and the genotypes GSTM1*0 and GSTT1*0 with published data. The frequencies of CYP1A1*2A, CYP2D6*4, CYP2E*5B, and GSTP1*B, genotyped in 311 unrelated persons (622 chromosomes) in both samples, were compared via the analysis of the molecular variance (ARLEQUIN 3.1) according to Excoffier *et al.* (1992). The fixation index (Fst) was estimated for the entire sample.

The two groups studied were in Hardy-Weinberg equilibrium with regard to genotype frequencies of the genes CYP1A1*2A, CYP2D6*4, CYP2E*5B and GSTP1*B. The mutant allele and null genotype frequencies found in the present study were compared with others described in the literature from both non-Brazilian and Brazilian populations (data presented in Tables 1  and 2). When our data were compared with literature data from non-Brazilian Afro-descendants, the frequencies of individuals with mutant alleles for the genes CYP2D6*4, GSTP1*B and null genotype GSTM1*0 were not homogeneously distributed between the populations of this study (Table 1). We believe that this discrepancy is due to the different methods used for the classification of ethnic origin among research groups, in spite of the parental population from North and South America may have different gene frequencies. In this sense is important to notice that the partial χ^2^ values from our sample were the main responsible for the observed significance. On the other hand, the frequencies of individuals with mutant alleles and null genotypes (GSTM1*0 and GSTT1*0) for the genes studied were homogeneously distributed between populations when the non-Brazilian Europeans and Euro-descendants were considered (Table 1). The frequencies of individuals with mutant alleles and null genotypes in Brazil, both for Afro-and Euro-descendants were homogeneously distributed (Table 2).

In the comparison of our groups we noticed that there was a homogeneous distribution of the frequency of the genotypes GSTM1*0 and GSTT1*0 between the Afro-descendants and Euro-descendants; the differences of the frequencies of individuals with dominant and recessive genotypes, respectively, were statistically not significant (χ^2^_1_ = 2.52; p ± 0.10 and χ^2^_1_ = 1.97; p > 0.10). The analysis of molecular variance (AMOVA) for the genes CYP1A1*2A, CYP2D6*4, CYP2E1*5B, and GSTP1*B showed that 97.47% of the component of genetic variance is present within the ethnic groups and 2.53% (p < 10^-4^) between them. This lower value justify the lower value of the fixation index or co-ancestry coefficient (Fst = 0.02508 and 0.02565 for Afro- and Euro-descendants, respectively, and 0.02529 for the entire group) observed in this study. F_st,_ is computed as a measure of the population division effect and values up to 0.05 indicate negligible genetic differentiation (Adeyemo *et al.*, 2005).

Biometabolism genes have been widely used in association studies, and they have contributed to the improvement in understanding the genetic basis of quantitative features (*e.g.*, susceptibility to complex diseases and drug response). Such studies must consider the impact of the population stratification and miscegenation degree of the control population (Ardlie *et al.*, 2002; Freedman *et al.*, 2004) in order to prevent false associations (Zembrzuski *et al.*, 2006). When genes with ethnic variation frequencies are evaluated in association studies (especially in complex diseases with multiple environmental and genetic factors), the high-risk group may present a low prevalence of the high-risk allele if other genetic or environmental risk factors predominate in that group (Ziv and Burchard, 2003).

The present report provides data that can contribute to the general profile of frequency and population dynamics of biometabolizing genes in groups of the Southern Brazilian population. These data constitute a valuable resource for the planning of future association studies in complex diseases like cancers.

## Figures and Tables

**Table 1:** Comparison between the present data and frequencies obtained in non-Brazilian samples.

Genes	Frequencies	n	Reference (population)		Frequencies	n	Reference (population)
	*African and Afro-descendants*				*European and Euro-descendants*		
CYP1A1*2A	0.210 0.239 0.235 0.301 ± 0.310	389 461 550 196	Le Marchand *et al.* 1998 (Hawaii and California - USA) Garte *et al.* 2001 (Africans - GSEC*) Wrensch *et al.* 2005 (San Francisco - USA) Present study χ^2^_3_ = 6.07; p > 0.10		0.094 0.104 0.092 0.104 0.106 0.152 ± 0.279	4453 453 419 520 146 115	Garte *et al.* 2001 (Europeans - GSEC*) Hung *et al.* 2003 (Europeans and Euro - Americans) Taioli *et al.* 2003 (Europeans and Euro - descendants (GSEC*) Raimondi *et al.* 2005 (Europeans - GSEC*) Wenzlaff *et al.* 2005 (Detroit - USA) Present study χ^2^_5_ = 5.32; p > 0.10

CYP2D6*4	0.071 0.070 0.078 0.054 0.145 ± 0.263	246 386 308 502 196	Leathart *et al.* 1998 (Los Angeles - USA) Huang *et al.* 1999 (Ghana) Wan *et al.* 2001 (Souhern California - USA) Gaedigk *et al.* 2002 (Atlanta - USA) Present study χ^2^_4_ = 16.98;p < 0.01		0.197 0.180 0.153 0.202 0.138 0.215 ± 0.249	211 408 360 305 105 114	Longuemaux *et al.* 1999 (France) Gaedigk *et al.* 2002 (Atlanta - USA) Scordo *et al.* 2004 (Italy) Gajecka *et al.* 2005 (Poland) Menoyo *et al.* 2006 (Spain) Present study χ^2^_5_ = 5.34; p > 0.30

CYP2E1*5B	0.070 0.079 ± 0.197	114l 196	Wu *et al.* 1997 (Texas - USA) Present study χ^2^_1_ = 0.086; p > 0.70		0.037 0.028 0.050 ± 0.152	1454 323 109	Garte *et al.* 2001 (Europeans - GSEC*) Gajecka *et al.* 2005 (Poland) Present study χ^2^_2_ = 1.36; p > 0.50

GSTM1*0	0.200 0.278 0.267 0.330 0.368 ± 0.480	120 259 479 114 190	Ford *et al.* 2000 (Columbia and New York - USA) Millikan *et al.* 2000 (North Carolina - USA) Garte *et al.* 2001 (Africans - GSEC*) Dandara *et al.* 2002 (Tanzania) Present study χ^2^_4_ = 13.03; p < 0.05		0.452 0.520 0.542 0.500 0.513 0.461 ± 0.500	168 369 395 1282 1981 115	Olshan *et al.* 2000 (North Carolina - USA) Millikan *et al.* 2000 (North Carolina - USA) Gudmundsdottir *et al.* 2001 (Iceland) Taioli *et al.* 2003 (GSEC*) Raimondi *et al.* 2005 (GSEC*) Present study χ^2^_5_ = 5.78; p > 0.30

GSTT1*0	0.166 0.250 0.242 ± 0.424	259 114 €190	Millikan *et al.* 2000 (North Carolina - USA) Dandara *et al.* 2002 (Tanzania) Present study χ^2^_2_ = 5.55; p > 0.05		0.130 0.164 0.132 0.174 ± 0.381	168 373 478 115	Olshan *et al.* 2000 (North Carolina - USA) Millikan *et al.* 2000 (North Carolina - USA) Mitrunen *et al.* 2001 (Finnish) Present study χ^2^_3_ = 3.01; p > 0.30

GSTP1*B	0.508 0.378 ± 0.332	247 196	Millikan *et al.* 2000 (North Carolina - USA) Present study χ^2^_1_ = 7.53; p < 0.01		0.310 0.306 0.288 0.259 0.291 0.283 ± 0.339	189 368 1138 481 153 115	Longuemaux *et al.* 1999 (France) Millikan *et al.* 2000 (North Carolina - USA) Garte *et al.* 2001 (Europeans - GSEC*) Mitrunen *et al.* 2001 (Finnish) Dufour *et al.* 2005 (Italy) Present study χ^2^_5_ = 2.99; p > 0.70

n = number of individuals; *GSEC - Genetic Susceptibility to Environmental Carcinogens Database.

**Table 2:** Comparison between the present data and frequencies obtained in other Brazilian samples

Genes	Frequencies	n	Authors (Brazilian region)		Frequencies	n	Authors (Brazilian region)
	*Afro-descendants*				*Euro-descendants*		
CYP1A1*2A	0.305 0.301 ± 0.310	100 196	Kvitko *et al.* 2006 (South) Present study χ^2^_1_ = 0.005;p > 0.90		0.106 0.173 0.152 ± 0.279	85 90 115	Torresan *et al.*, 2008 (South) Kvitko *et al.* 2006 (South) Present study χ^2^_2_ = 1.64; p > 0.30

CYP2E1*5B	0.029 0.058 0.079 ± 0.197	136 86 196	Gattás *et al.* 2000 (Southeast) Rossini *et al.* 2006 (Southeast) Present study χ^2^_2_ = 3.71;p > 0.10		0.069 0.061 0.050 ± 0.152l	151 66 109	Rossini *et al.* 2006 (Southeast) Torresan *et al.*, 2008 (South) Present study χ^2^_2_ = 0.40; p > 0.80

CYP2D6*4*	-	-	-		0.188 0.215 ± 0.249	85 114	Torresan *et al.*, 2008 (South) Present study χ^2^_1_ = 0.21; p > 0.50

GSTM1*0	0.330 0.342 0.328 0.340 0.368 ± 0.480	117 272 137 100 190	Arruda *et al.* 1998 (Northeast) Rossini *et al.* 2002 (Southeast) Gattás *et al.* 2004 (Southeast) Kvitko *et al.* 2006 (South) Present study χ^2^_4_ = 0.72; p > 0.90		0.450 0.489 0.446 0.500 0.463 0.461 ± 0.500	130 319 233 90 95 115	Arruda *et al.* 1998 (Southeast) Rossini *et al.* 2002 (Southeast) Gattás *et al.* 2004 (Southeast) Kvitko *et al.* 2006 (South) Torresan *et al.*, 2008 (South) Present study χ^2^_5_ = 1.65; p > 0,80

GSTT1*0	0.190 0.257 0.263 0.280 0.242 ± 0.424	117 272 137 100 190	Arruda *et al.* 1998 (Northeast) Rossini *et al.* 2002 (Southeast) Gattás *et al.* 2004 (Southeast) Kvitko *et al.* 2006 (South) Present study χ^2^_4_ = 3.14; p > 0.50		0.185 0.215 0.223 0.211 0.295 0.174 ± 0.381	130 319 233 90 95 115	Arruda *et al.* 1998 (Southeast) Rossini *et al.* 2002 (Southeast) Gattás *et al.* 2004 (Southeast) Kvitko *et al.* 2006 (South) Torresan *et al.*, 2008 (South) Present study χ^2^_5_ = 5.53; p > 0,20

GSTP1*B	0.420 0.378 ± 0.332	100 196	Kvitko *et al.* 2006 (South) Present study χ^2^_1_ = 0.50; p > 0.30		0.315 0.278 0.330 0.283 ± 0.339	319 90 85 115	Rossini *et al.* 2002 (Southeast) Kvitko *et al.* 2006 (South) Torresan *et al.*, 2008 (South) Present study χ^2^_3_ = 0.99;p > 0.80

n = number of individuals; * no data to compare.
